# A novel immune signature predicts immunotherapy responsiveness and reveals the landscape of the tumor immune microenvironment in head and neck squamous cell carcinoma

**DOI:** 10.3389/fgene.2022.1051051

**Published:** 2022-11-11

**Authors:** Qiwei Wang, Yinan Zhao, Fang Wang, Guolin Tan

**Affiliations:** ^1^ Department of Otolaryngology Head and Neck Surgery, Third Xiangya Hospital, Central South University, Changsha, China; ^2^ Xiangya School of Nursing, Central South University, Changsha, China; ^3^ Department of Otolaryngology, University Hospital Rechts der Isar, Technical University of Munich, Munich, Germany

**Keywords:** HNSCC, immune signature, immunotherapy, prognosis, tumor immune microenvironment

## Abstract

**Background:** Immune-checkpoint blockade (ICB) has been routinely implemented to treat head and neck squamous cell carcinoma (HNSCC) patients. However, only a few patients benefit from immune checkpoint inhibitor (ICI) therapies.

**Methods:** In this study, we used a combined cohort (including the GSE41613, GSE65858, TCGA, and CELL cohorts) to identify hub genes significantly associated with ICB and activated CD8^+^ T-cell gene signatures. We performed single‐sample gene set enrichment analysis (ssGSEA) to quantify the expression of hub genes; we then constructed a novel immune signature named “the IMS” that can predict immunotherapy responsiveness, prognosis, immune infiltration, and clinical characteristics. Data from the GSE102349 external cohort and the pembrolizumab cohort obtained from a clinical trial were used to validate the efficiency of the IMS. In addition, we revealed potential mechanisms of the antitumor response by analyzing the HNSCC single-cell database. Finally, we used the LASSO algorithm to build an IMS-related risk model.

**Results:** The high IMS group was associated with significant immune activation, better prognosis, and increased immunotherapy responsiveness; thus, the IMS potentially represents a candidate biomarker for ICB. Moreover, a tumor microenvironment with a higher IMS underwent remarkable metabolic reprogramming characterized by enrichment in the glycolysis/gluconeogenesis, oxidative phosphorylation, and citrate cycle (TCA cycle) pathways. We also revealed key information on cellular crosstalk between the IMS and other immune lineages, which may mechanistically explain immune escape. In addition, we constructed and validated a risk prediction model (CD2, TBC1D10C, and CD3E) that could stratify HNSCC patients based on survival and response to ICB treatment.

**Conclusion:** IMS is a signature closely correlated with the tumor immune microenvironment. The findings of this study contribute to the understanding of the immune landscape in HNSCC patients. IMS may aid in the clinical management of HNSCC patients through the identification of effective immunotherapies for specific patients.

## Introduction

Head and neck squamous cell carcinoma (HNSCC) is the sixth most prevalent cancer type worldwide; more than 890,000 people were diagnosed with HNSCC in 2018 (Bray et al., 2018). In the past decade, immune checkpoint inhibitor (ICI) treatment has been verified as providing stable clinical benefits to patients with advanced cancers, including HNSCC. For example, by blocking the PD-1 signaling receptor, the tumor-specific CD8^+^ T lymphocytes in the tumor microenvironment (TME) restore cytotoxicity, thereby inhibiting tumor immune escape ability and controlling the disease. However, a clinical trial revealed that, in patients beyond tumor control, only a few HNSCC patients (18%) benefit from ICI treatment (Chow LQM, 2020). Hence, it is imperative to identify and quantify potential effective biomarkers and signaling pathways of HNSCC to improve our understanding of the immune biology environment.

Many studies have benefitted from large, multi-dimensional common datasets—such as The Cancer Genome Atlas (TCGA)—and have confirmed that the infiltration level of immune cells and alterations in cancer genomics are correlated with the immune checkpoint blockade (ICB) response. For example, a higher level of CD8 T cells is strongly associated with longer survival and increased sensitivity to anti-PD-1 monoclonal antibody therapy (Cristescu et al., 2018). Cytotoxic CD8 T cells play a key role in eradicating malignant cells and can provide long-term protective immunity. Therefore, exploring potential immunotherapeutic signatures based on the ICB and CD8 gene sets could represent a reliable strategy for classifying patients who might be responsive to ICIs.

Here, by analyzing bulk transcriptomics and single-cell RNA sequencing, we identify a novel immune signature (IMS) associated with patients’ response to ICIs. We unveiled specific molecular mechanisms and identified hub genes to better understand anti-tumor biology. Our findings highlight the potential immunotherapy targets and pathways in HNSCC.

## Methods

### HNSCC dataset source and processing

We summarized the activated CD8^+^ T-cell transcriptome gene set reported by Charoentong et al. (Charoentong et al., 2017) (Supplementary Table S3). The ICB gene set and HNSCC immunotherapy cohort were obtained from Cindy Yang et al. (Cindy Yang et al., 2021) (Supplementary Table S4). In addition, our research integrated data from TCGA (expression profiling by high-throughput sequencing), the Gene Expression Omnibus databases GSE65858 (expression profiling by array) and GSE41613 (expression profiling by array), and CELL (Huang et al., 2021) (expression profiling by high-throughput sequencing) database. Although these four databases contain different sequencing data, the R software package “combat” could remove the batch effects from different experiment types and platforms. Hence, we used this package and filtered common genes to construct a combined cohort. The Gene Expression Omnibus database GSE102349 cohort and the pembrolizumab (Cindy Yang et al., 2021) cohort from a clinical trial were used for external validation.

### Weighted gene co-expression network construction and hub gene identification

Weighted gene co-expression network analysis (WGCNA) was performed using the WGCNA R package (Langfelder and Horvath, 2008). The best pick soft threshold value was 4; the Pearson’s method was used to calculate the correlation among ICB gene set, activated CD8^+^ T-cell gene set, and modules. The gene modules with the lowest *p* value in the immune checkpoint blockade and activated CD8^+^ T-cell modules were selected as candidate gene modules related to immune checkpoint inhibitors. We identified candidate genes based on the correlation value and sorted the array in descending order. Hub genes were filtered according to the following criteria: MM (correlation between gene module and activated CD8 gene set), GS (correlation between gene module and immune checkpoint gene set), and GS1 (correlation between activated CD8 and immune checkpoint gene set) > 0.8.

### Hypergeometric analysis of hub genes function and pathway enrichment

We used the clusterProfiler R package (Yu et al., 2012) to perform Kyoto Encyclopedia of Genes and Genomes (KEGG) analysis of the hub genes. Gene Ontology (GO) analysis, including biological process (BP), molecular function (MF), and cellular component (CC), was performed in the same manner. Adjusted *p* < 0.05 was used to determine the significance of the biological functions and pathways of the hub genes.

### Construction of molecular types based on the hub genes

We used the consensus clustering algorithm in R named “ClassDiscovery” to distinguish hub gene expression patterns. Single‐sample gene set enrichment analysis (ssGSEA) and univariate Cox regression methods were used to construct a novel immune signature, named “the IMS.”

### Estimation of immune infiltration and ICI response

The ssGSEA, CIBERSORT (Newman et al., 2015), and “ESTIMATE” methods were employed to evaluate the absolute abundance of multiple immune cell populations and calculate the immune score. Gene sets related to immune checkpoint blockade and sensitivity to immunotherapy were associated with the IMS and could predict the ICI response.

### Single-cell quality control and data processing

We downloaded the GSE139324 single-cell cohort from the Gene Expression Omnibus database. We used the R package Seurat (Butler et al., 2018) to analyze this cohort. We filtered the sample with <10% mitochondrial genes. We used the FindVariableGenes function to select highly variable genes with parameter nfeatures = 2000. These variable genes were used as inputs for PCA using the RunPCA function. Dims = 1:15 was used for the FindNeighbors function, and resolution = 0.5 were used for the FindClusters function. Thus, 12 clusters were identified, and cluster analysis was performed using the RunUMAP function. We used the FindAllMarkers function to identify differentially expressed genes (DEGs) for each cluster with the parameters min.pct = 0.25 and thresh.use = 0.25. We compared hub genes (CD96, CD247, CD3G, SH2D1A, TBC1D10C, CXCR3, SIRPG, SLA2, and ARHGAP9) in DEGs for IMS annotation in clusters. The Single R package was used to annotate the remaining clusters. We used the MuSic deconvolution method (Wang et al., 2019) to estimate the IMS proportion in TCGA bulk-seq. The CellChat method (Jin et al., 2021) was used to construct cellular communication. We used the scMetabolism method (Wu et al., 2022a) to perform metabolism quantification for the IMS; the metabolism signaling pathway gene set was downloaded from the Molecular Signatures Database (MSigDB) (Liberzon et al., 2015) hallmark gene set collection.

### Construction and verification of the prognostic model

We used LASSO regression to filter optimal prognostic gene combinations to classify our combined cohort. The risk score formula was generated using the Predict function in R. According to the stratification risk patterns, the “survival” R package was employed to determine the demarcation point of each dataset of each subgroup, and the “survminer” R package plotted Kaplan–Meier curves. All patients were classified into high- or low-risk groups based on the median cut-off value. We also validated these results in the external GSE102349 and pembrolizumab (Cindy Yang et al., 2021) cohorts from a clinical trial.

### Statistical analysis

All statistical analysis and bioinformatics methods were performed using R (V4.1.2, https://www.r-project.org/). Correlation analysis was conducted using the Pearson and Spearman methods. The Wilcoxon test was performed to compare continuous variables and ordered categorical variables.

### Data and code availability statements

All datasets used in this study are available in a public database. The codes supporting the conclusions of this article can be obtained by reasonable request to the corresponding author.

## Results

### Research process

**FIGURE 1:**
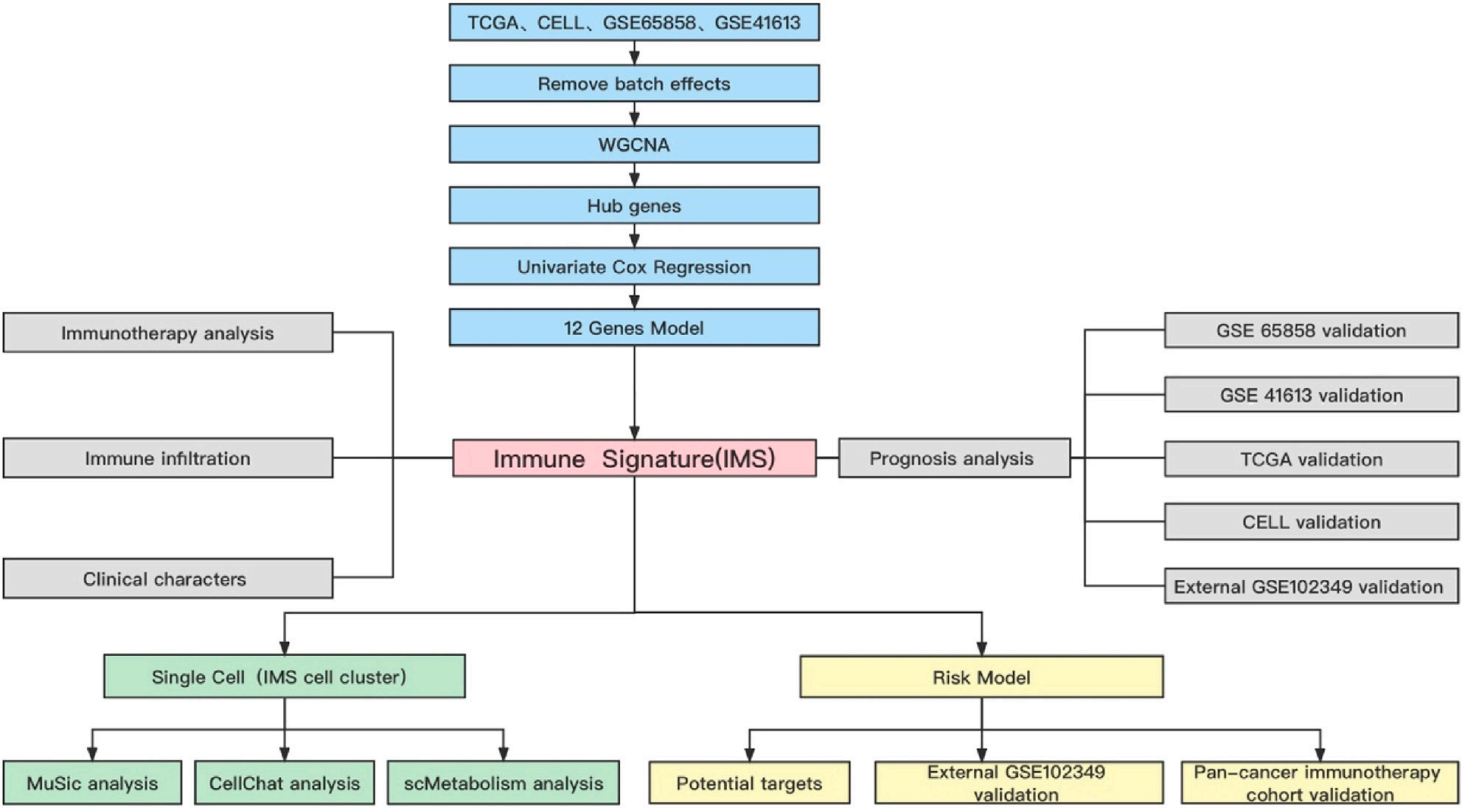
Flow chart of this study.

**FIGURE 2:**
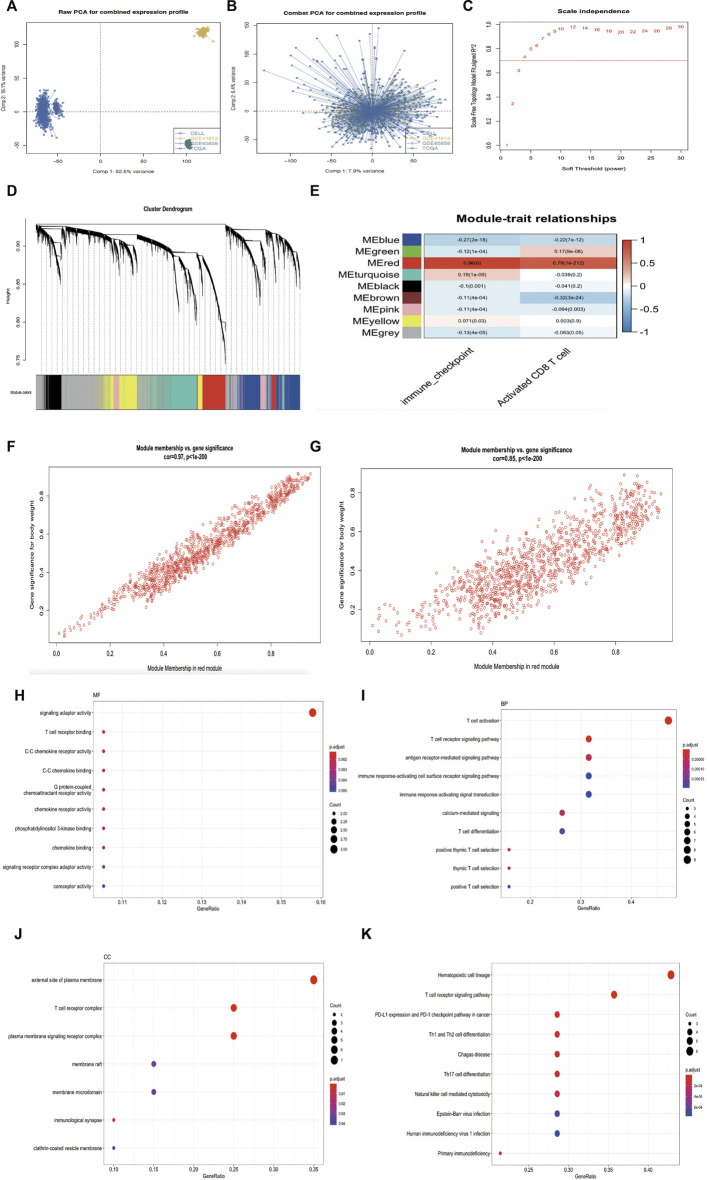
(Continued). Research process. **(A)** Principal component analysis showed the gene expression profile in four HNSCC cohorts (GSE65858, GSE41613, TCGA, and CELL database) before elimination of the batch effects. **(B)** Principal component analysis showed the gene expression profile in four HNSCC cohorts (GSE65858, GSE41613, TCGA and CELL database) after elimination of the batch effects. **(C)** Analysis of network topology for various soft-thresholding powers. The red line indicates best pick soft threshold value = 4. **(D)** Cluster dendrogram of the differentially expressed genes based on different metrics. Each color indicates a single module of weighted co-expressed genes. **(E)** Correlation heatmap between the red module and activated CD8 T-cell and immune checkpoint signatures in combined cohort. Every column includes the concordance value and *p* value. **(F–G)** Correlation scatter map in both immune checkpoint signature **(F)** and activated CD8 T-cell signature of red module **(G)**. **(H–J)** MF, BP, and CC analysis of 20 hub genes. **(K)** KEGG pathway enrichment analysis for 20 hub genes.

A flowchart of this study of IMS-related characteristics associated with HNSCC is provided in Figure 1. First, we used the “combat” software package to avoid batch effects. The gene expression profile of each cohort was dispersive (Figure 2A); after the “combat” process, the profile was agminated (Figure 2B). The ICB and activated CD8^+^ T-cell gene sets were obtained from Charoentong et al. (Charoentong et al., 2017) and Cindy Yang et al. (Cindy Yang et al., 2021). We filtered out genes that exhibited less variance than all quartiles of variance in the integrated cohort samples to construct WGCNA (Langfelder and Horvath, 2008) and identify key modules. Using the selection method described above, 13,048 genes were obtained from 977 samples. We used these genes to build nine different colored cluster dendrograms based on the best pick soft threshold value (Figure 2C) and found that the red gene module was extremely positively correlated with activated CD8 T-cell and immune checkpoint signatures (activated CD8 T cell: *r* = 0.78, *p* = 6e–105; immune checkpoint: *r* = 0.93, *p* = 7e–221, Figures 2D,E). We further applied the method of correlation analysis to create a plot and found a significant correlation between red module members and the gene signature of activated CD8 T cells and immune checkpoints (immune checkpoint: *r* = 0.97, *p* < 1e–200; activated CD8 T cell: *r* = 0.85, p < 1e–200; Figures 2F,G). The above result indicated that the red module genes play an important role in responsiveness to HNSCC immunotherapy. Therefore, we extracted those genes in the red module, calculated their corresponding correlation value, sorted the array in a descending order, and filtered both MM, GS, and GS1 >0.8 as hub genes (Table 1).

**TABLE 1:** 20 Hub genes.

moduleGenes	MM	GS	GS1
CD48	0.926604886	0.800746447	0.900384012
ARHGAP9	0.917185835	0.80250068	0.893224992
CD2	0.902078578	0.853224821	0.914141467
CORO1A	0.895081873	0.810303791	0.883376826
SH2D1A	0.885400282	0.831343766	0.881920263
TBC1D10C	0.878388208	0.837079858	0.870648173
CD3E	0.876696	0.828357429	0.883652564
HSCT	0.875260547	0.823555945	0.859493958
CD3G	0.872087532	0.82483128	0.887972451
CD247	0.871033865	0.83890792	0.881848041
CD3D	0.870528592	0.886309137	0.895750187
SLA2	0.867521398	0.846417413	0.883378874
SIRPG	0.861009041	0.846457191	0.882788945
CXCR3	0.860076796	0.806477374	0.864782986
CD96	0.850736777	0.807605878	0.854626303
GZMK	0.834441905	0.805893185	0.821888253
CXCR6	0.832592868	0.811703704	0.851306327
NKG7	0.827653932	0.890920351	0.860515962
CD7	0.820449395	0.816523291	0.841903385
CD8A	0.816318943	0.845573165	0.850149829

Then, we used the “clusterProfiler” package (Yu et al., 2012) in R to analyze the hub gene enrichment landscape (Figures 2H–K). GO analysis showed that these genes were mainly enriched in functions such as T-cell receptor binding, T-cell activation, T-cell differentiation, and the external side of the plasma membrane. KEGG analysis showed that hub genes were associated with the PD-1 signaling pathway in cancer, the T-cell receptor signaling pathway, and T-cell differentiation. These enrichment results indicated that hub genes support biological functions in T-cell regulation and the immune response (Spangler et al., 2015; Joseph et al., 2020) and may provide the basis for a novel classification of immunophenotypes in head and neck squamous cell carcinoma.

### The IMS could predict HNSCC patient survival and HPV status

**FIGURE 3:**
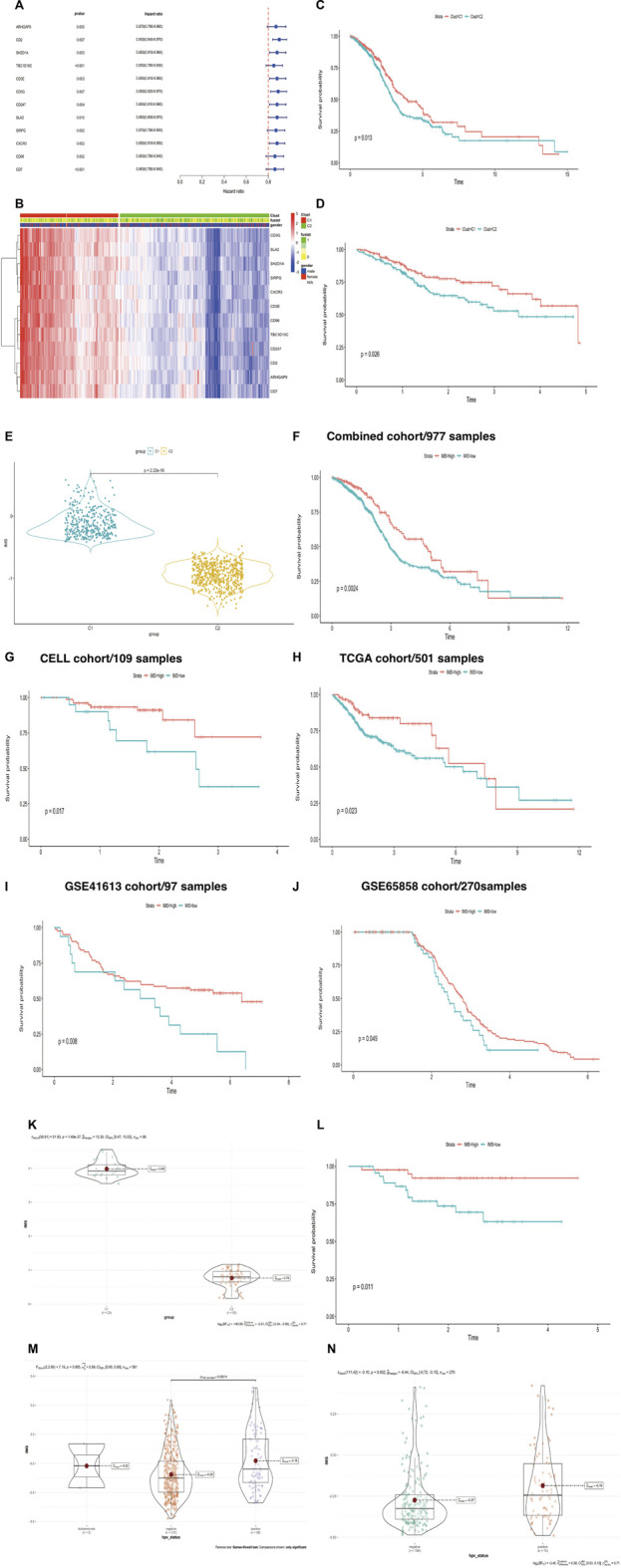
(Continued). Construction of IMS could predict HNSCC patient survival and HPV status. **(A)** Univariate Cox regression analysis of about 12 genes. Hazard ratio (HR) < 1 represents that these genes were protective factors. **(B)** The heatmap displays the correlation between the two types of 12 genes and the expression variance, C1 (387 cases), C2 (589 cases), “1” means dead, “0” means alive, “fustat” means survival status. **(C,D)** The Kaplan-Meier plot exhibited significant statistic *p* value of overall survival rate among the two phenotypes of 12 genes in the combined (log rank *p* = 0.013) and TCGA cohorts (log rank *p* = 0.026), respectively. C1 was better than C2, unit of time (years). **(E)** Violin plot showed differential IMS expression in the C1 and C2 groups; *p*-value<2.22e-16. **(F–J)** The Kaplan-Meier plot exhibited significant statistical *p* value of overall survival rate among the two IMS phenotypes in the combined cohort (log rank *p* = 0.0024), CELL cohort (log rank *p* = 0.017), TCGA cohort (log rank *p* = 0.023), GSE41613 (log rank *p* = 0.008), and GSE65858 cohort (log rank *p* = 0.049), respectively, unit of time (years). **(K)** IMS in groups of GSE102349 cohort; high IMS group represents C1, low IMS group represents C2; *p* = 1.49e-37. **(L)** The Kaplan–Meier plot exhibited significant statistical *p* value of overall survival rate among the two IMS phenotypes in the external GSE102349 cohort. Unit of time (years). **(M)** IMS in the group of TCGA cohort; HPV-negative group (410), HPV-positive group (89); *p* = 0.00014. **(N)** IMS in the group of GSE65858 cohort; HPV-negative group (196), HPV-positive group (74); *p* = 0.002.

To assess whether hub genes could predict HNSCC patient survival, we used the univariate Cox regression method to filter candidate prognostic genes, including CD2, SH2D1A, TBC1D10C, CD3E, CD3G, CD247, SLA2, SIRPG, CXCR3, CD96, CD7, and ARHGAP9 (Figure 3A). We used the R package “ClassDiscovery” to classify two unique modification patterns and named them Clust_C1 (387 samples) and Clust_C2 (589 samples, Figure 3B). After removing samples with incomplete clinical data, we plotted the survival curve between these two subtypes. Clust_C1 provides a particularly significant survival advantage, and Clust_C2 is associated with poor prognosis (log rank *p* = 0.013, Figure 3C). In the internal cohort (TCGA), this modification pattern also revealed that Clust_C1 exhibits longer survival than Clust_C2 (log rank *p* = 0.026, Figure 3D).

We then used ssGSEA to quantify the expression of these 12 genes, which were used to construct the IMS. The violin plot showed that the IMS was significantly higher in C1 than in C2 (Figure 3E, *p* value < 2.22e-16). Using the optimal cut-off value determined with the R package “survminer,” the Kaplan‒Meier curve showed that the IMS was not only a prognostic factor for head and neck squamous cell carcinoma in this combined cohort but also in the individual cohorts (Figures 3F–J, log rank *p* = 0.0024, 0.017, 0.023, 0.008, and 0.049 for the combined cohort, CELL cohort, TCGA cohort, GSE41613 cohort, and GSE65858 cohort, respectively).

In the external cohort GSE102349, we used the same method to quantify the expression of these 12 genes and found that these 12 immune genes related to the IMS also classified the patients and predicted significantly favorable survival (Figures 3K,L; *p*-value = 1.49e-37, log rank *p* = 0.011). A previous study demonstrated that patients with HPV-positive HNSCC have better overall survival than those with HPV-negative HNSCC (Ang et al., 2010). Therefore, we also detected the IMS in the HPV-positive and HPV-negative groups. The results showed that the IMS in the HPV-positive group was remarkably higher than that in the HPV-negative group in the TCGA and GSE65858 cohorts (*p* = 0.000165; *p* = 0.002; Figures 3M,N). Those results showed that HPV + HNSCC patients with longer survival could be due to a high IMS level.

### IMS could predict immunotherapy responsiveness and classify HNSCC patients based on immunophenotype

**FIGURE 4:**
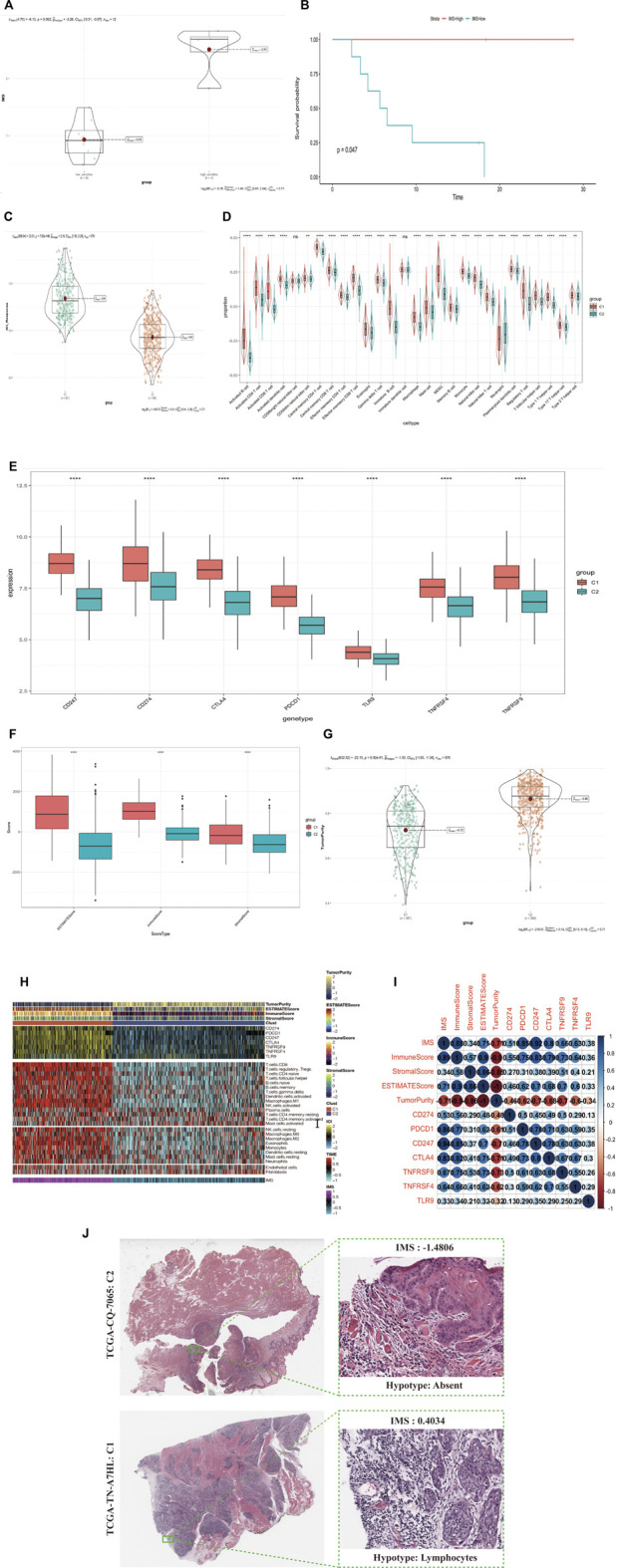
(Continued). IMS could predict immunotherapy response and stratify the immunophenotype in HNSCC patients. **(A)** IMS in group of immunotherapy cohort; low-sensitivity group, high-sensitivity group; *p* = 0.002. **(B)** The Kaplan-Meier plot exhibited a statistical *p* value of overall survival rate among the two IMS phenotypes in the immunotherapy cohort. Unit of time (months). **(C)** IMS in groups of combined cohorts; high IMS group (387) represents Clust_C1, low IMS group (589) represents Clust_C2; *p* = 7.56e-146. **(D)** Enrichment of each immune cell type infiltrating in group of Clust; C1 (387 cases), C2 (589 cases); combined cohort; the asterisk represents the different *p* values (* <0.05; ** <0.01; *** <0.001, **** <0.0001). **(E)** Differential expression of immune checkpoint genes (CD247, CD274, CTLA4, PDCD1, TLR9, TNFRSF4, TNFRSF9) in group of Clust; C1 (387 cases) and C2 (589 cases); combined cohort; asterisks represent different *p* values (* <0.05; ** <0.01; *** <0.001, **** <0.0001). **(F)** ESTIMATEScore, ImmuneScore, and StromalScore in group of Clust; C1 (387 cases), C2 (589 cases); from combined cohort; asterisks represent different *p* values (* <0.05; ** <0.01; *** <0.001, **** <0.0001). **(G)** TumorPurity in group of Clust; C1 (387 cases), C2 (589 cases); combined cohort; *p*-value = 6.92e-81. **(H)** Complex-heatmap displays the landscape in the combined cohort; top panel displays the expression of genes involved in immune checkpoint targets; bottom panel displays the infiltration level of 24 microenvironment cell types. ESTIMATEScore, ImmuneScore, StromalScore, TumorPurity, C1 (387 cases), and C2 (589 cases) are labeled at top of heatmap, IMS are labeled at the bottom of the heatmap. **(I)** Bubble plot displays the correlation between the IMS, four score type, and seven immune checkpoint target genes. Blue means a positive correlation, red means a negative correlation, color depth and color size means the intensity of the correlation. The levels of correlation are marked with numbers. Upper triangular matrix represents Pearson correlation, lower triangular matrix represents Spearman correlation. **(J)** Image representing the pathological HE staining variation between the high and low IMS groups (TCGA database).

In the HNSCC ICI treatment cohort (Cindy Yang et al., 2021), we found that the group highly sensitive to the ICI response had a significantly higher IMS than the low-sensitivity group (*p* = 0.002; Figure 4A). We divided the immunotherapy cohort into two groups based on the IMS and found that the high IMS group had a longer survival time (Figure 4B, log rank *p* = 0.047). We then calculated the ICI response score as described by Wu et al. (Wu et al., 2022b). A higher score represented greater sensitivity to immune checkpoint inhibitor treatment; we found that the high IMS group in C1 had a remarkably higher score than that in C2 (Figure 4C; *p* = 7.56e-146).

To detect the relationship between the immune cell-type infiltration and IMS, we divided samples from these combined cohorts into the C1 and C2 groups. We used the ssGSEA method to standardize the immune cell signatures obtained from Bindea et al. (Bindea et al., 2013). We found that the infiltration levels of every immune cell type in the C1 group were significantly higher than those in the C2 group, except for CD56^bright^ natural killer cells and immature dendritic cells (Figure 4D). ICB therapy is effective for HNSCC, so we collected seven immune checkpoint target genes (CD247, CD274, PDCD1, TNFRSF9, TNFRSF4, CTLA4, and TLR9) reported in previous studies (Ramos-Casals et al., 2020; van de Donk et al., 2021). We found that all seven of these genes exhibited significant differential expression between the high and low IMS groups (Figure 4E).

We used the ESTIMATE R package to quantify the scores of stromal and immune cells in this combined cohort: the ESTIMATEScore, ImmuneScore, StromalScore, and TumorPurity. We found that the ESTIMATEScore, ImmuneScore, and StromalScore were higher in the C1 group than the C2 group, but that the C1 group had lower TumorPurity (Figures 4F,G; *p* = 6.92e-81). We also used the CIBERSORT algorithm (Newman et al., 2015) to calculate the infiltration of different immune cell types in these groups and found that the C1 group had a significantly higher infiltration level. We plotted a combined heatmap to display the above results (Figure 4H) and found that the IMS was positively correlated with the ESTIMATEScore, ImmuneScore, StromalScore, and seven immune checkpoint target genes but was negatively correlated with TumorPurity (Figure 4I). We further confirmed that Clust_C1 exhibited greater levels of immune cell infiltration, but Clust_C2 had less infiltration of immune cells in the tumor nests (HNSCC TCGA Pathology cohort; Figure 4J).

### Exploration of IMS characteristics using the single-cell RNA sequencing database

**FIGURE 5:**
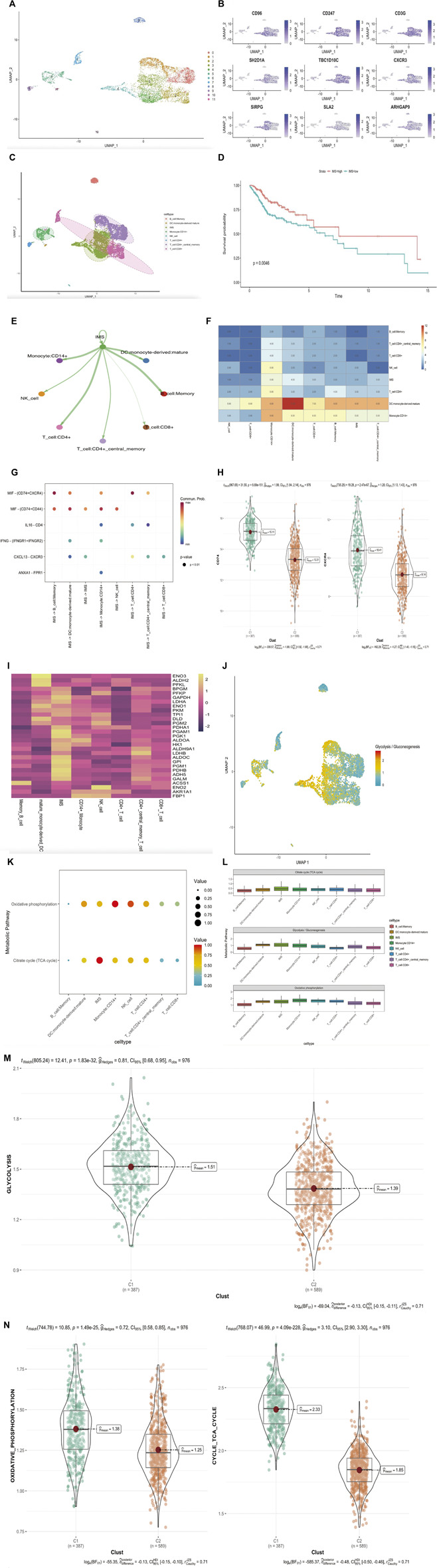
(Continued). Exploration of IMS characteristic in single-cell RNA sequencing database. **(A)** UMAP plot of selected 6435 single cells in immune cells (CD45 positive). Different colors represent different cell types. **(B)** UMAP plot shows the expression of nine genes in IMS classifier. **(C)** UMAP plot of selected 6435 single cells in immune cells (CD45 positive). Twelve cell clusters were divided into eight cell types. **(D)** Kaplan–Meier plot displays significant differences of survival rate among high-IMS proportion and low-IMS proportion in TCGA cohorts. MuSic Deconvolution method. High group was more favorable than low group, unit of time (years). **(E)** The differential immune–immune cellular communication weight coefficient shows IMS cross-talk between all immune cell type. **(F)** The heatmap of immune–immune cellular communication shows the counts of IMS cross-talk between all immune cell types. **(G)** Communication network of the significant ligand–receptor pairs between IMS and other immune cell types, which contribute to the signaling from IMS to memory B cell, dendritic cell, CD14^+^ monocyte cell, CD4^+^ T cell, CD8^+^ T cell, CD4^+^ memory T cell, and NK cell subpopulations. Dot color reflects communication probabilities and dot size represents computed *p*-values. Empty space means the communication probability is zero. *p*-Values are computed from a one-sided permutation test. **(H)** CD74 and CXCR4 (MIF) in groups of combined cohorts; high pathway group (387) represents high IMS, low pathway group (589) represents low IMS; *p* = 8.69e-151, *p* = 2.47e-67. **(I)** Heatmap of metabolic genes’ average expression in different immune cell types. **(J)** Glycolysis/gluconeogenesis pathway distribution plot of all immune cells. **(K)** Enrichment of immune cell types in oxidative phosphorylation and TCA cycle pathways. Dot color reflects enrichment probabilities and dot size represents computed *p*-values. **(L)** The rank of each immune cell-type enrichment in glycolysis/gluconeogenesis, oxidative phosphorylation, and TCA cycle pathways. The result shows IMS ranked first in the glycolysis/ gluconeogenesis and citrate cycle (TCA cycle) pathway but fifth in the oxidative phosphorylation pathway. **(M)** Glycolysis/gluconeogenesis pathway in groups of combined cohorts; high pathway group (387) represents high IMS, low pathway group (589) represents low IMS; *p* = 1.8e-32. **(N)** Oxidative phosphorylation and TCA cycle pathway in groups of combined cohorts; high pathway group (387) represents high IMS, low pathway group (589) represents low IMS; *p* = 1.49e-25, *p* = 4.09e-228.

We selected CD45-positive cells as immune cells to elucidate the tumor immune microenvironment of HNSCC and identified 6435 cells from three patients after quality control. We distinguished 12 distinct clusters based on a resolution value of 0.5 (Figure 5A). IMS clusters were annotated using nine genes from the IMS classifier (CD96, CD247, CD3G, SH2D1A, TBC1D10C, CXCR3, SIRPG, SLA2, and ARHGAP9; Figure 5B). According to the cell cluster distribution and classifier gene co-expression regions, we labeled Clusters 3, 4, and 6 as the IMS cluster. We used the Single R package to classify several other distinct clusters: B-cell memory cells, NK cells, mature monocyte-derived DCs, CD14^+^ monocytes, CD4^+^ central memory T cells, CD8^+^ T cells, and CD4^+^ T cells (Figure 5C). In addition, we performed the MuSic deconvolution method (Wang et al., 2019) to calculate the bulk tissue proportion of IMS in the TCGA cohort with this single-cell RNA sequencing database reference (Supplementary Table S1). As we had expected, HNSCC patients with a high IMS had a remarkably favorable survival (log rank *p* = 0.0046; Figure 5D). This result validated the IMS constructed by ssGSEA or by MuSic deconvolution as a prognostic indicator in HNSCC.

To further detect the enrichment of IMS populations in HNSCC immune cells, we hypothesized that IMS populations might be functionally distinct across other immune cell types. Hence, we performed ligand–receptor-based immune–immune cellular cross-talk analysis (Jin et al., 2021) (Figure 5E) and generated a heatmap to better assess the frequency of immune–immune cellular cross-talk (Figure 5F). These results suggested that HNSCC immune cells could be preferentially reprogrammed by the impact of TME, thereby inducing their specific functional status—likely explained by the intrinsic difference in differential gene expression. To distinguish the significant ligand–receptor interactions of the IMS with other immune cell types, we used the same method (Jin et al., 2021) to study the signaling of the intercellular communication network in HNSCC immune cells. We identified macrophage migration inhibitory factor (MIF) ligand‒receptor pairs (CD74+CXCR4 and CD74^+^CD44) as the most significant signaling pathway that facilitates communication between the IMS and every immune cell type except CD8^+^ T cells (Figure 5G). In the combined bulk cohort, the high IMS group also exhibited high expression of CD74 and CXCR4 (Figure 5H; *p* = 8.69e-151, *p* = 2.47e-67); therefore, these ligand–receptor pairs specifically enriched in HNSCC immune cell types may provide a clue for targeted immunotherapy.

Cellular glucose metabolism plays a determinant role in immune cell function and viability. Some investigations revealed that upregulation of glycolysis/gluconeogenesis, the tricarboxylic acid cycle (TCA cycle), and oxidative phosphorylation were hallmarks of antitumor immune cell activation (Wang et al., 2011; Menk et al., 2018; Patel et al., 2019). Thus, we used the scMetabolism method (Wu et al., 2022a) to better understand these three metabolic pathways in HNSCC immune cells. First, we detected the average expression of glucose metabolic genes in different T-cell types; all immune cells showed a strong imbalanced distribution of metabolic genes associated with glycolysis/gluconeogenesis signaling genes (Figures 5I,J). We subsequently calculated the enrichment abundance of two other metabolism-associated pathways in different immune cell types and found an IMS, indicating extensive involvement in the TCA cycle and oxidative phosphorylation compared to all immune cell types (Figure 5K). The boxplot of IMS enrichment revealed that the TCA cycle and glycolysis/gluconeogenesis metabolic pathways ranked first among all immune cell types and that the oxidative phosphorylation pathway ranked fifth (Figure 5L). Our combined cohort further verified that glycolysis/gluconeogenesis, the TCA cycle, and oxidative metabolic pathways were dominant in the IMS high group (Figure 5M,N; *p* = 1.8e-32, *p* = 1.49e-25, and *p* = 4.09e-228, respectively). These results revealed that cellular energy metabolic regulation could mediate the phenotype and function of IMS cells in response to antitumor effects.

### Construction and verification of the IMS risk prediction model

**FIGURE 6:**
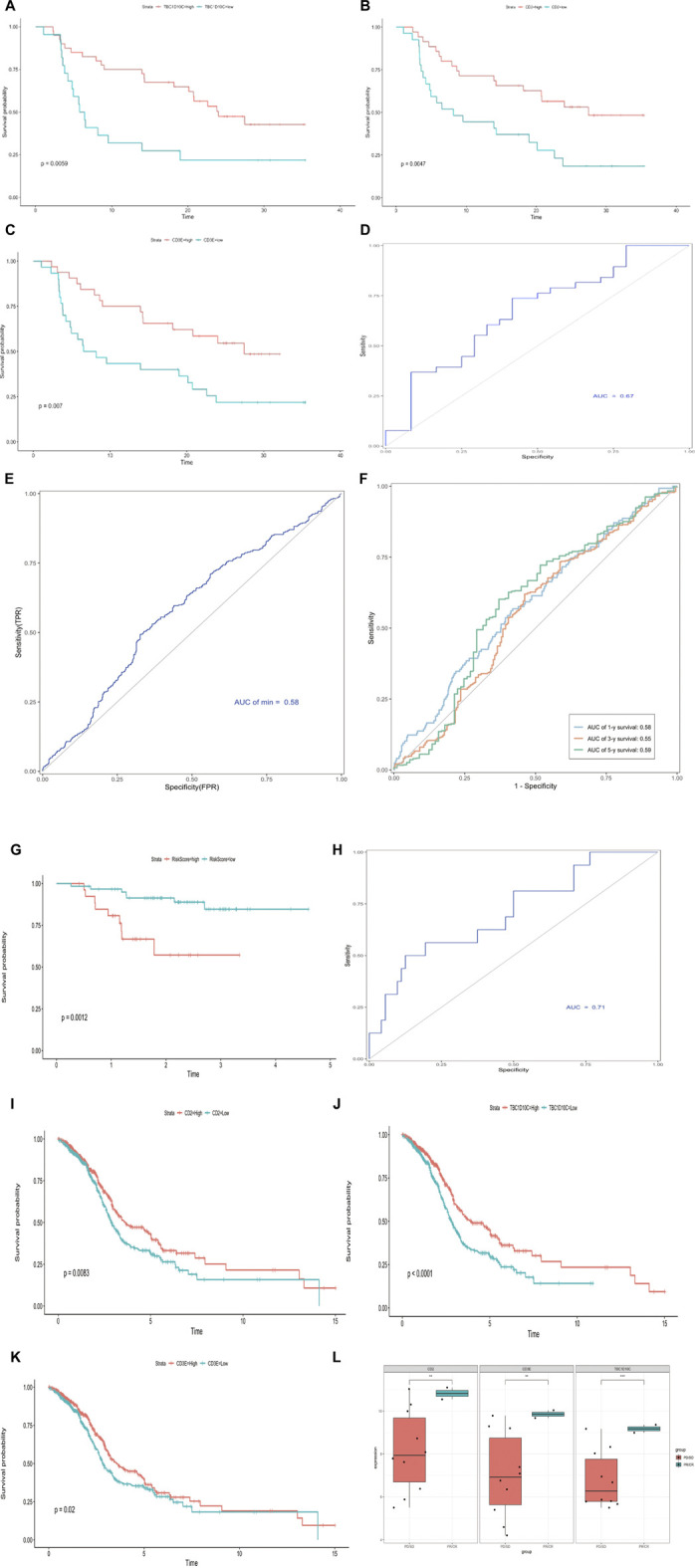
Construction and verification of IMS risk prediction model. **(A)** Hundred‐time cross‐validation for tuning parameter selection in the LASSO model; combined cohort. **(B)** Risk score range in two groups (0 = alive, 1 = dead); combined cohort. **(C)** Kaplan–Meier curve shows high-risk and low-risk groups based on the risk score; combined cohort; log rank *p* < 0.0001; unit of time (years). **(D)** Kaplan–Meier curve shows high-risk and low-risk groups based on the risk score; TCGA cohort; log rank *p* = 0.00054, unit of time (years). **(E)** Receiver operating characteristic curve in combined cohort; AUC = 0.58. **(F)** Time–ROC curve in combined cohort; AUC 1 year = 0.58; AUC 3 years = 0.55; AUC 5 years = 0.59. **(G)** Kaplan–Meier curve shows high-risk and low-risk groups based on the risk score; external GSE102349 cohort; log rank *p* = 0.0012; unit of time (years). **(H)** Receiver operating characteristic curve in GSE102349 cohort; AUC = 0.71. **(I–K)** Kaplan–Meier curve shows high-risk and low-risk groups based on CD2, TBC1D10C, and CD3E expression, respectively; combined cohort CD2 log rank *p* = 0.0083; TBC1D10C log rank *p* < 0.0001; CD3E log rank *p* = 0.02; unit of time (years). **(L)** CD2, TBC1D10C, and CD3E expression in immunotherapy cohort, complete response (CR), partial response (PR), stable disease (SD), progressive disease (PD) as per RECIST criteria; asterisks represent different *p* values (* <0.05; ** <0.01; *** <0.001, **** <0.0001).

Several studies have validated that immune-related molecules are biomarkers for prognosis (Fridman et al., 2017; Bruni et al., 2020; Zitvogel et al., 2021). Thus, we used the LASSO algorithm to filter candidate immune genes from the IMS classifier (Figure 6A). Three immune genes (CD2, CD3E, and TBC1D10C) were identified using the lambda-min value, with one immune gene (TBC1D10C) identified using lambda-1se value. Considering the precision of future clinical testing, we selected three immune genes to construct a risk model. We used the “Predict” function in R to calculate the risk score based on these three genes and classified this combined cohort based on the median risk score. The box chart showed that the risk score in the alive and dead groups was significantly different. We found that the dead group had an exceedingly higher risk score than the alive group (p = 2e-5; Figure 6B). Kaplan–Meier analysis results showed that the high-score group had significantly higher mortality than the low-score group; this finding was validated in the internal TCGA cohort (log rank *p* < 0.0001, log rank *p* = 0.00054; Figures 6C,D). A receiver operating characteristic (ROC) curve was used to validate the sensitivity and specificity of this risk model; we found that the AUC of the combined cohort risk model was 0.58 (Figure 6E). We also calculated the AUC values at 1, 3, and 5 years (1-year AUC = 0.58, 3-year AUC = 0.55, 5-year AUC = 0.59; Figure 6F). These values suggested that the risk model based on the combined cohort exhibited predictive significance. We also validated these results in the external cohort GSE102349 (log rank *p* = 0.0012, AUC = 0.71; Figures 6G,H). Then, we divided HNSCC patients into two groups according to the expression of these three immune genes. The Kaplan–Meier analysis results showed that patients with high expression of these genes had significantly better survival than those with low expression in the combined cohort (CD2, log rank *p* = 0.0083, TBC1D10C, log rank *p* < 0.0001, CD3E, log rank *p* = 0.02; Figures 6I–K). In addition, we analyzed the expression of these three immune genes in patients in the HNSCC immunotherapy cohort with complete response (CR), partial response (PR), stable disease (SD), and progressive disease (PD), as defined by RECIST criteria. We found significantly increased expression of all these genes in CR/PR patients compared with SD/PD patients (Figure 6L).

Furthermore, we analyzed CD2, TBC1D10C, and CD3E expression in patients enrolled in a Phase II basket clinical trial of pembrolizumab (Cindy Yang et al., 2021). This clinical trial assessed a pan cancer immunotherapy cohort (including HNSCC, breast cancer, ovarian cancer, and melanoma). As expected, the Kaplan–Meier curve showed that these three genes were prognostic factors (Supplementary Figures S6A–C; CD3E, log rank *p* = 0.007, CD2, log rank *p* = 0.0047, TBC1D10C, log rank *p* = 0.0059). The ROC results obtained by multivariate Cox regression validated the predictive value of these three genes in the pan cancer immunotherapy cohort (Supplementary Figure S6D; AUC = 0.67).

## Discussion

Our study identified potential immunotherapy biomarkers by analyzing the gene expression profiles from the combined and immunotherapy cohorts. Screening hub genes with significant MM, GS, and GS1 values greater than 0.8, 20 genes among them were particularly prominent. All these genes were enriched in the functions of immunotherapy and immune response. We classified HNSCC patients using these hub genes and constructed a novel immune signature named the IMS to calculate survival rates and performed immune enrichment analyses. We found that a high IMS predicts longer survival and abundant immune infiltration. These results indicate that the high IMS group will benefit from ICI treatment. These results were validated using the external GSE102349 cohort and HNSCC immunotherapy cohort. According to Topalian, S. L et al. (Topalian et al., 2012), better ICI efficacy was significantly correlated with a higher expression of related immune checkpoint genes, such as PDCD1. Another study showed that HPV-negative head and neck tumor patients exhibit poor prognoses compared to HPV-positive patients (Johnson et al., 2020). In our study, the high IMS group exhibits a higher expression of checkpoint molecules compared with the low IMS group. In addition, a high IMS indicated that HNSCC patients were more likely to have an HPV-positive status. Our study also found a meaningfully positive correlation between the IMS and immune checkpoint targets. Those observations indicate that HPV+ HNSCC patients are more likely to benefit from immunotherapy. Moreover, our study revealed that the IMS was positively correlated with the ESTIMATEScore, ImmuneScore, and StromalScore, but negatively correlated with TumorPurity, hence giving us an expanded understanding that the IMS might exert a positive impact on clinical outcomes.

Single-cell RNA sequencing revealed a complex immune microenvironment in head and neck squamous tumors. We classified patients based on the IMS and seven immune cell clusters, and the deconvolution results showed that a high IMS proportion was robustly related to favorable survival in the TCGA cohort. These results suggest that these IMS classifier genes could be potentially used to guide clinical immunotherapy treatment. Energy metabolism is essential for the antitumor function of immune effector cells. T-cell replication and function are highly dependent on the upregulation of specific glycolytic programs, including aerobic glycolysis, the hexosamine biosynthesis pathway (HBP), the pentose phosphate pathway (PPP), and the TCA cycle (Leone and Powell, 2020). For example, the PPP metabolizes glucose-6-phosphate to generate NADPH and ribose-5-phosphate, which are required for fatty acid and plasma membrane synthesis in newly activated CD8^+^ T cells. In addition, the inhibition of 2-oxoglutarate-dependent dioxygenases through alterations in TCA metabolites such as αKG, succinate, and fumarate increase memory cell differentiation in CD8^+^ T cells (Kidani et al., 2013; Tyrakis et al., 2016). Our study revealed that the IMS cell cluster was particularly enriched in these metabolic pathways, which is consistent with our combined bulk sample-based results. These results suggest that IMS cells in HNSCC have undergone extensive remodeling and are strongly enriched in metabolic pathways, indicating that metabolism pathways or genes could regulate immune checkpoint targets. To this end, the combination of metabolic drugs with immune checkpoint inhibitors represents a promising method of enhancing the efficacy of immune checkpoint blockade.

A comprehensive investigation of intercellular communications is essential for understanding the interactions and spatial proximity among HNSCC immune cells. In our study, we first identified MIF ligand‒receptor pairs as the dominant signaling pathway that facilitate communication between IMS cells and other immune cell types. This MIF ligand‒receptor analysis of the putative interactions displayed here can be pursued further to better understand the ecosystem cultivated by intercellular communication in the HNSCC tumor microenvironment. Sumaiya et al. (Sumaiya et al., 2022) reported that MIF was overexpressed in almost all types of solid tumors, including HNSCC, and induced negative impacts on the immune system, thus leading to tumor growth and metastasis. Our study further contextualizes this finding for the combined bulk cohort, thus providing an explanation for the poor response rate of ICI treatment in HNSCC. In summary, our immune signature IMS can be useful in characterizing the HNSCC tumor immune microenvironment, stratifying HNSCC patients into different immunophenotype groups, predicting the prognosis of HNSCC patients, and promoting an understanding of the mechanism underlying the antitumor response and immune escape in HNSCC.

We constructed a prognostic model based on the IMS, with the validation results showing that the risk model exhibited high accuracy and sensitivity. Moreover, the risk score can be used as an independent prognostic factor, indicating that it has a stable and powerful survival predictive ability. The effectiveness and rationality of establishing the IMS-related risk model based on a big data algorithm will facilitate the clinical diagnosis and treatment process in patients. Previous studies have verified that CD3E, CD2, and TBC1D10C play a significant role in immune activation and cytotoxicity. For example, CD3E is part of the T-cell receptor/CD3 complex (TCR/CD3 complex) and plays a role in T-cell development and signal transduction, which is essential for the activation and positive selection of CD4 or CD8 T cells (Doucey et al., 2003; Fischer et al., 2005). CD2 is implicated in the activation of T cells by promoting adhesion and T-cell receptor signaling, and the upregulation of CD2 could enhance antitumor T-cell responses (Demetriou et al., 2020). In a recent study, TBC1D10C was reported to be a regulator of immune activity and to play an important role in shaping macrophage activity by remodeling the cytoskeleton-plasma membrane to facilitate different T-cell functions (Villagomez et al., 2021). At present, no studies have demonstrated the correlation between these three immune genes and immunotherapy in HNSCC. We used clustering analysis to confirm that these three immune genes were more highly expressed in the high IMS group. In addition, the classification of patients in our combined bulk cohort based on risk score and comparison of gene expression in the CR/PR and SD/PD groups suggested that CD3E, CD2, and TBC1D10C represent genes that are potentially predictive of response to immunotherapy. We found that each of the three immune genes was associated with good survival in both the cohort from the immunotherapy clinical trial and the combined cohort. These results confirmed that CD3E, CD2, and TBC1D10C could be used independently as genes that predict response to immunotherapy.

Despite these promising findings, we recognize some limitations of our research. For example, fresh clinical sample collection is difficult, so we did not conduct external validation using fresh tumor samples; further experimental evidence from cellular and molecular assays is thus needed to validate the findings of this study. In addition, we conducted a retrospective cohort study with a commonly used internet database, so the results should be further verified in a multicenter prospective cohort study. Moreover, the tumor immune environment includes multiple immune populations, and patient prognosis depends on CD8 cells as well as CD4 cells, Treg cells, and myeloid cells, including macrophages, neutrophils, and myeloid-derived suppressor cells. We will focus more on molecular interactions between these immune cells in our follow-up research.

## Conclusion

We established a novel and robust immune signature referred to as “the IMS” to classify immunophenotypes in head and neck squamous cell carcinoma (HNSCC) patients; this signature was validated using internal and external cohorts. Responsiveness to immunotherapy was predicted for different IMS groups, and this information may provide an important foundational framework for exploring HNSCC immunotherapy targets. We identified IMS cell clusters in a single-cell database, suggesting that a high IMS predicts favorable survival based on cross-talk between IMS and other immune lineages. We observed unique possibilities to target metabolic pathways to enhance the immunotherapy response. In addition, we constructed a prognostic model based on the IMS and provided reliable biomarkers of prognosis in HNSCC patients. Overall, our study contributes to the understanding of the tumor immune landscape in patients with HNSCC and serves as a basis for future in-depth exploration of the role of IMS cells.

## Data Availability

The original contributions presented in the study are included in the article/Supplementary Material; further inquiries can be directed to the corresponding author.

## Abbreviations

CELL, a public HNSCC cohort from Huang C et al.; CR, complete response; DEGs, differentially expressed genes; GEO, Gene Expression Omnibus; GEP, gene expression profile; GO, Gene Ontology; HBP, hexosamine biosynthesis pathway; HNSCC, head and neck squamous cell carcinoma; GS, correlation between gene module and immune checkpoint gene set; GS1, correlation between activated CD8 and immune checkpoint gene set; ICB, immune checkpoint blockade; ICIs, immune checkpoint inhibitors; IMS, immune signature; KEGG, Kyoto Encyclopedia of Genes and Genomes; MIF, macrophage migration inhibitory factor; MM, correlation between gene module and activated CD8 gene set; PD, progressive disease; PPP, pentose phosphate pathway; PR, partial response; SD, stable disease; TCGA, The Cancer Genome Atlas; TME, the tumor microenvironment; WGCNA, weighted gene co-expression network analysis.
