# Supplementary material for: A novel immune signature predicts immunotherapy responsiveness and reveals the landscape of the tumor immune microenvironment in head and neck squamous cell carcinoma
Source: Front Genet. 2022 Nov 11;13:1051051. doi: 10.3389/fgene.2022.1051051 (PMC9691887; doi:10.3389/fgene.2022.1051051)
Supplement: Supplementary file 2 [file Table1.docx]

**Supplementary Table1: MuSic deconvolution result**

|  | **IMS** | **T_cell:CD8+** |
| --- | --- | --- |
| TCGA-D6-6515 | 9.06E-05 | 9.93E-07 |
| TCGA-CV-7180 | 0.00032298 | 6.53E-06 |
| TCGA-CV-7183 | 0.00072437 | 0.000130075 |
| TCGA-CV-A6JN | 0.00061199 | 6.21E-05 |
| TCGA-CQ-7069 | 0.00022885 | 2.35E-06 |
| TCGA-CQ-6219 | 2.84E-05 | 2.43E-06 |
| TCGA-IQ-A6SH | 9.24E-05 | 2.00E-05 |
| TCGA-F7-A61V | 7.42E-05 | 1.04E-06 |
| TCGA-HD-A6I0 | 0.0005087 | 1.48E-06 |
| TCGA-CN-6992 | 6.39E-05 | 4.26E-07 |
| TCGA-BA-5556 | 0.00013021 | 3.18E-05 |
| TCGA-CV-7245 | 0.00012455 | 2.33E-05 |
| TCGA-UF-A7JV | 0.00019765 | 3.74E-05 |
| TCGA-CV-5440 | 6.17E-05 | 1.20E-05 |
| TCGA-UF-A7JO | 7.42E-06 | 4.81E-07 |
| TCGA-CV-5443 | 0.00070426 | 0.000297849 |
| TCGA-P3-A5Q5 | 0.00021967 | 4.36E-05 |
| TCGA-CN-A498 | 0.00016207 | 6.09E-07 |
| TCGA-CX-7082 | 8.23E-05 | 1.36E-05 |
| TCGA-CV-7432 | 0.00010205 | 3.28E-07 |
| TCGA-CV-7248 | 3.50E-05 | 6.48E-06 |
| TCGA-KU-A66T | 5.42E-05 | 1.87E-06 |
| TCGA-P3-A5Q6 | 1.76E-05 | 8.69E-07 |
| TCGA-CN-5374 | 2.29E-05 | 2.55E-06 |
| TCGA-IQ-A61J | 0.00036744 | 9.27E-06 |
| TCGA-CV-A45Y | 7.65E-05 | 2.35E-05 |
| TCGA-P3-A6T0 | 0.00023165 | 1.54E-05 |
| TCGA-CN-4740 | 0.00024608 | 3.39E-05 |
| TCGA-CN-5370 | 2.93E-05 | 7.85E-07 |
| TCGA-CN-6024 | 0.0002258 | 1.61E-05 |
| TCGA-CR-7397 | 0.00012038 | 2.74E-05 |
| TCGA-QK-AA3J | 3.07E-05 | 1.73E-07 |
| TCGA-BA-A6DI | 0.00020471 | 2.63E-05 |
| TCGA-CV-7413 | 9.07E-05 | 1.07E-05 |
| TCGA-CR-7377 | 7.05E-05 | 8.04E-07 |
| TCGA-D6-6823 | 9.07E-05 | 8.27E-07 |
| TCGA-CV-7253 | 6.43E-05 | 1.58E-05 |
| TCGA-CV-7238 | 0.00016278 | 1.80E-05 |
| TCGA-CV-6942 | 6.80E-05 | 7.29E-06 |
| TCGA-BA-A6DA | 0.00057983 | 3.85E-05 |
| TCGA-MT-A67D | 1.92E-05 | 7.30E-07 |
| TCGA-CQ-5323 | 0.00045846 | 0.000117947 |
| TCGA-CR-7390 | 0.00020143 | 1.53E-05 |
| TCGA-UF-A7JK | 0.0001739 | 1.83E-05 |
| TCGA-DQ-7591 | 0.00011471 | 1.53E-07 |
| TCGA-CN-5358 | 9.98E-05 | 7.96E-06 |
| TCGA-CV-6945 | 7.74E-05 | 9.41E-06 |
| TCGA-CQ-6218 | 0.00031595 | 2.92E-05 |
| TCGA-CV-6943 | 0.00014121 | 2.11E-05 |
| TCGA-CN-4737 | 0.00025323 | 5.05E-05 |
| TCGA-QK-A6V9 | 0.000279 | 3.88E-05 |
| TCGA-CR-6472 | 9.97E-05 | 1.49E-05 |
| TCGA-UF-A7JH | 0.0001849 | 9.41E-06 |
| TCGA-BA-4077 | 2.96E-05 | 7.03E-07 |
| TCGA-CV-7411 | 0.00138173 | 0.000173301 |
| TCGA-MT-A67A | 0.00055256 | 5.72E-05 |
| TCGA-F7-8489 | 0.00021372 | 8.36E-07 |
| TCGA-DQ-5625 | 3.59E-05 | 5.07E-06 |
| TCGA-CV-6950 | 0.00014977 | 2.76E-05 |
| TCGA-P3-A6T7 | 0.00013189 | 3.09E-05 |
| TCGA-UF-A718 | 0.00030411 | 5.88E-05 |
| TCGA-QK-A6VB | 3.94E-05 | 4.15E-06 |
| TCGA-CV-6938 | 5.59E-05 | 5.25E-06 |
| TCGA-CV-7250 | 5.53E-05 | 8.45E-06 |
| TCGA-CV-6959 | 9.61E-05 | 1.71E-05 |
| TCGA-HD-7832 | 7.61E-05 | 1.97E-05 |
| TCGA-CV-A45X | 8.88E-05 | 2.51E-05 |
| TCGA-CN-A6UY | 2.38E-05 | 3.99E-07 |
| TCGA-CQ-6222 | 0.00013548 | 3.02E-05 |
| TCGA-MZ-A7D7 | 9.06E-05 | 6.36E-06 |
| TCGA-CQ-5329 | 8.93E-05 | 1.69E-06 |
| TCGA-CV-5973 | 0.00024974 | 6.86E-07 |
| TCGA-BA-5555 | 0.00010406 | 5.06E-06 |
| TCGA-CV-6935 | 7.94E-05 | 2.71E-07 |
| TCGA-HD-8635 | 9.08E-05 | 3.43E-05 |
| TCGA-CR-6487 | 2.70E-05 | 6.86E-06 |
| TCGA-CN-6018 | 5.92E-05 | 1.10E-07 |
| TCGA-CR-6484 | 0.00039988 | 0.000106983 |
| TCGA-CV-7425 | 0.0001603 | 1.99E-05 |
| TCGA-BB-A5HU | 4.49E-05 | 2.99E-07 |
| TCGA-HD-7831 | 0.00013672 | 5.30E-06 |
| TCGA-CR-7364 | 0.00089524 | 0.000297578 |
| TCGA-CN-A63W | 5.08E-05 | 8.58E-06 |
| TCGA-D6-A6EK | 0.00047217 | 2.45E-05 |
| TCGA-BA-5557 | 0.00020822 | 3.09E-05 |
| TCGA-BA-6869 | 4.90E-05 | 2.16E-06 |
| TCGA-CR-6477 | 0.00017077 | 7.83E-07 |
| TCGA-UF-A71B | 0.0002871 | 4.53E-05 |
| TCGA-H7-A6C5 | 0.00029803 | 1.51E-05 |
| TCGA-CV-6933 | 0.00023766 | 1.91E-05 |
| TCGA-QK-A6IF | 3.85E-05 | 7.71E-08 |
| TCGA-DQ-7588 | 0.00027059 | 1.69E-05 |
| TCGA-BA-6873 | 0.00016861 | 1.19E-05 |
| TCGA-CV-5442 | 8.39E-05 | 5.85E-06 |
| TCGA-CR-7392 | 8.27E-05 | 1.27E-06 |
| TCGA-UF-A71A | 6.41E-05 | 1.50E-06 |
| TCGA-CN-A642 | 3.82E-05 | 6.96E-06 |
| TCGA-CV-A45U | 4.76E-05 | 1.41E-05 |
| TCGA-CN-6012 | 8.19E-05 | 2.24E-05 |
| TCGA-CV-A45W | 0.00017685 | 8.85E-05 |
| TCGA-CV-A6K0 | 6.95E-05 | 1.39E-05 |
| TCGA-CN-6022 | 3.62E-05 | 6.53E-06 |
| TCGA-CN-A6V3 | 0.00024696 | 2.21E-05 |
| TCGA-QK-A8Z7 | 1.99E-05 | 9.51E-07 |
| TCGA-CN-A497 | 2.81E-05 | 8.81E-07 |
| TCGA-F7-A50G | 0.00029913 | 3.18E-05 |
| TCGA-IQ-A61I | 4.14E-05 | 3.77E-08 |
| TCGA-CR-7373 | 9.48E-05 | 7.54E-07 |
| TCGA-CN-A63T | 5.44E-05 | 1.14E-06 |
| TCGA-CN-6019 | 9.17E-05 | 1.19E-05 |
| TCGA-CR-7386 | 0.00051765 | 4.22E-06 |
| TCGA-HD-A6HZ | 3.47E-05 | 8.69E-08 |
| TCGA-T3-A92M | 0.00031736 | 5.81E-05 |
| TCGA-IQ-7631 | 0.00014932 | 8.22E-06 |
| TCGA-CR-7398 | 3.93E-05 | 5.44E-06 |
| TCGA-UF-A71E | 8.39E-05 | 1.41E-05 |
| TCGA-UF-A719 | 3.14E-05 | 7.70E-06 |
| TCGA-BA-A4IF | 3.90E-05 | 1.46E-06 |
| TCGA-CV-6960 | 0.00016947 | 2.53E-05 |
| TCGA-CN-4725 | 0.00021586 | 1.32E-05 |
| TCGA-WA-A7H4 | 5.19E-05 | 1.20E-05 |
| TCGA-CV-7103 | 8.86E-05 | 6.18E-06 |
| TCGA-CV-5441 | 7.04E-05 | 2.52E-07 |
| TCGA-CR-7367 | 7.88E-05 | 1.24E-05 |
| TCGA-CR-5249 | 3.21E-05 | 2.06E-06 |
| TCGA-F7-A620 | 4.99E-05 | 3.18E-07 |
| TCGA-QK-A8Z9 | 5.98E-05 | 2.28E-06 |
| TCGA-IQ-7630 | 4.74E-05 | 1.10E-05 |
| TCGA-CN-4738 | 7.06E-05 | 8.34E-06 |
| TCGA-CQ-5331 | 0.00025898 | 3.11E-05 |
| TCGA-CV-7418 | 0.00010224 | 1.11E-05 |
| TCGA-BA-A6D8 | 7.45E-05 | 6.63E-06 |
| TCGA-CV-5971 | 6.78E-05 | 6.71E-06 |
| TCGA-CQ-6220 | 0.00017046 | 5.73E-05 |
| TCGA-QK-A8ZB | 0.00011189 | 1.51E-05 |
| TCGA-CR-6481 | 2.36E-05 | 3.23E-06 |
| TCGA-BB-8596 | 1.82E-05 | 3.74E-07 |
| TCGA-UF-A71D | 4.33E-05 | 6.36E-06 |
| TCGA-CV-7437 | 0.00016785 | 1.68E-05 |
| TCGA-CV-A464 | 0.0001591 | 2.60E-07 |
| TCGA-CV-A6JD | 1.37E-05 | 1.54E-06 |
| TCGA-CQ-7071 | 0.00013694 | 5.94E-06 |
| TCGA-CV-6962 | 0.00010055 | 3.07E-05 |
| TCGA-CN-5366 | 8.42E-05 | 1.80E-06 |
| TCGA-CN-4723 | 6.27E-05 | 1.79E-07 |
| TCGA-F7-A624 | 2.76E-05 | 6.02E-08 |
| TCGA-CV-A463 | 0.00010064 | 2.96E-05 |
| TCGA-CV-7423 | 0.00017315 | 4.49E-06 |
| TCGA-BB-4228 | 5.81E-05 | 4.31E-07 |
| TCGA-CV-6953 | 0.00013054 | 1.40E-05 |
| TCGA-CV-7261 | 5.14E-05 | 5.90E-06 |
| TCGA-P3-A5QF | 7.74E-05 | 6.94E-06 |
| TCGA-CR-6470 | 0.00054303 | 0.000154895 |
| TCGA-CV-5979 | 0.00017326 | 1.65E-05 |
| TCGA-CR-6471 | 0.00024223 | 5.85E-05 |
| TCGA-CV-7430 | 0.00028292 | 1.11E-05 |
| TCGA-CV-7099 | 8.48E-05 | 1.09E-05 |
| TCGA-CQ-6221 | 0.00031928 | 1.50E-05 |
| TCGA-QK-A6IJ | 0.00022301 | 2.65E-05 |
| TCGA-CV-7406 | 0.00018247 | 1.73E-05 |
| TCGA-CV-7435 | 0.00012137 | 6.32E-07 |
| TCGA-CV-7446 | 6.90E-05 | 7.94E-07 |
| TCGA-BA-6870 | 9.99E-05 | 2.37E-05 |
| TCGA-MZ-A5BI | 4.13E-05 | 7.80E-06 |
| TCGA-CR-6478 | 8.65E-05 | 1.42E-07 |
| TCGA-CN-6021 | 0.00028942 | 3.87E-06 |
| TCGA-CQ-6224 | 0.00014167 | 2.51E-05 |
| TCGA-HD-7754 | 0.00061907 | 9.66E-05 |
| TCGA-F7-A622 | 0.00039201 | 3.40E-06 |
| TCGA-P3-A6T2 | 7.08E-05 | 2.50E-06 |
| TCGA-CR-7383 | 7.51E-05 | 6.10E-06 |
| TCGA-CR-7379 | 0.00028977 | 3.07E-05 |
| TCGA-CV-7236 | 0.00016036 | 2.66E-06 |
| TCGA-CN-4727 | 3.74E-05 | 6.60E-06 |
| TCGA-CQ-5327 | 7.34E-05 | 3.78E-07 |
| TCGA-C9-A480 | 0.00047917 | 1.14E-05 |
| TCGA-CV-A6JO | 0.00020912 | 5.27E-06 |
| TCGA-CR-6492 | 7.54E-05 | 1.11E-07 |
| TCGA-BA-6872 | 0.00029349 | 1.88E-05 |
| TCGA-BB-4223 | 3.58E-05 | 1.18E-06 |
| TCGA-CV-A6K2 | 4.80E-05 | 1.40E-05 |
| TCGA-HD-8634 | 0.00021657 | 4.02E-05 |
| TCGA-CN-6994 | 0.00022179 | 4.68E-06 |
| TCGA-D6-A4Z9 | 0.00010547 | 2.88E-06 |
| TCGA-CV-7440 | 4.14E-05 | 8.47E-07 |
| TCGA-CR-7376 | 9.76E-05 | 3.98E-07 |
| TCGA-CR-7391 | 0.00019786 | 3.06E-05 |
| TCGA-T2-A6X2 | 0.00025888 | 6.67E-05 |
| TCGA-BA-4074 | 3.65E-05 | 2.59E-07 |
| TCGA-CN-4734 | 0.00045797 | 7.09E-06 |
| TCGA-CV-6441 | 2.22E-05 | 3.38E-06 |
| TCGA-CV-A6JY | 0.00013094 | 1.36E-05 |
| TCGA-D6-6825 | 0.00020851 | 3.21E-06 |
| TCGA-CR-5247 | 0.00011974 | 2.32E-05 |
| TCGA-CV-7410 | 0.00020681 | 3.28E-05 |
| TCGA-CV-7091 | 0.00013384 | 5.62E-06 |
| TCGA-BA-5153 | 0.00054782 | 8.10E-05 |
| TCGA-CV-7428 | 4.15E-05 | 4.46E-06 |
| TCGA-CX-7086 | 0.00013776 | 2.82E-05 |
| TCGA-CN-5364 | 4.90E-05 | 1.27E-07 |
| TCGA-BA-A6DB | 4.08E-05 | 7.65E-06 |
| TCGA-CV-7097 | 1.97E-05 | 3.43E-06 |
| TCGA-CQ-5325 | 0.0006883 | 0.000245531 |
| TCGA-H7-A6C4 | 0.00016351 | 2.05E-07 |
| TCGA-CN-4731 | 6.64E-05 | 1.99E-06 |
| TCGA-CV-7427 | 9.80E-05 | 1.61E-05 |
| TCGA-CR-6467 | 0.00038264 | 7.27E-05 |
| TCGA-BA-5151 | 0.00010294 | 2.31E-05 |
| TCGA-CV-7178 | 6.09E-05 | 3.46E-06 |
| TCGA-CV-A461 | 4.04E-05 | 9.56E-07 |
| TCGA-BA-5559 | 2.34E-05 | 1.42E-06 |
| TCGA-CR-6493 | 0.00011841 | 1.14E-05 |
| TCGA-CV-A6JT | 0.00037658 | 0.000129572 |
| TCGA-UF-A7JJ | 5.59E-05 | 1.12E-06 |
| TCGA-RS-A6TO | 0.00015491 | 2.50E-05 |
| TCGA-CR-7372 | 0.0003626 | 8.50E-05 |
| TCGA-D6-6827 | 1.73E-05 | 1.19E-06 |
| TCGA-QK-A6IH | 0.00029873 | 8.41E-05 |
| TCGA-CN-6011 | 8.63E-05 | 3.92E-06 |
| TCGA-CR-6474 | 7.51E-05 | 9.32E-06 |
| TCGA-CV-6939 | 1.19E-05 | 4.13E-08 |
| TCGA-D6-8568 | 9.97E-05 | 5.41E-06 |
| TCGA-HD-A634 | 5.41E-05 | 4.66E-06 |
| TCGA-UF-A7JD | 0.00012015 | 2.84E-07 |
| TCGA-CV-6951 | 4.32E-05 | 1.25E-05 |
| TCGA-F7-8298 | 5.22E-05 | 2.14E-06 |
| TCGA-CV-6941 | 9.62E-05 | 1.02E-05 |
| TCGA-H7-A76A | 0.00047091 | 0.000182896 |
| TCGA-D6-A6EP | 4.53E-05 | 6.62E-06 |
| TCGA-UF-A7JS | 0.00021731 | 1.85E-05 |
| TCGA-CV-A45Q | 0.00032411 | 8.22E-06 |
| TCGA-QK-A6VC | 3.01E-05 | 5.54E-07 |
| TCGA-BA-A6DL | 7.87E-05 | 1.07E-05 |
| TCGA-CR-7365 | 0.00034415 | 1.04E-06 |
| TCGA-CV-6934 | 0.00010277 | 3.09E-06 |
| TCGA-P3-A6SW | 6.88E-05 | 2.54E-05 |
| TCGA-HD-8314 | 0.00030983 | 2.60E-05 |
| TCGA-CN-4742 | 0.00035351 | 4.07E-05 |
| TCGA-IQ-A61H | 3.86E-05 | 4.99E-06 |
| TCGA-CX-7085 | 0.00107137 | 0.000438996 |
| TCGA-CV-7235 | 6.04E-05 | 1.61E-05 |
| TCGA-CV-7434 | 0.00010151 | 1.49E-05 |
| TCGA-CR-6480 | 7.50E-05 | 2.56E-07 |
| TCGA-BB-A6UM | 8.58E-05 | 1.17E-05 |
| TCGA-C9-A47Z | 0.00015102 | 7.03E-06 |
| TCGA-CR-7382 | 0.00011327 | 1.91E-05 |
| TCGA-BB-4225 | 2.21E-05 | 3.93E-07 |
| TCGA-BA-A4IG | 0.00010443 | 1.40E-05 |
| TCGA-CR-7393 | 8.06E-05 | 7.91E-06 |
| TCGA-T2-A6X0 | 0.00052787 | 0.000125551 |
| TCGA-F7-A623 | 8.05E-05 | 1.95E-07 |
| TCGA-IQ-7632 | 0.00024081 | 8.80E-07 |
| TCGA-QK-A652 | 0.00011881 | 9.68E-06 |
| TCGA-WA-A7GZ | 0.000186 | 1.26E-05 |
| TCGA-CR-7388 | 8.05E-05 | 2.44E-05 |
| TCGA-CV-A6K1 | 0.00013542 | 6.99E-06 |
| TCGA-CN-5363 | 5.87E-05 | 3.75E-06 |
| TCGA-CV-6956 | 6.30E-05 | 5.56E-06 |
| TCGA-CV-7263 | 6.68E-05 | 7.39E-07 |
| TCGA-CN-A49C | 0.00014789 | 1.44E-05 |
| TCGA-IQ-A6SG | 0.00015184 | 2.73E-05 |
| TCGA-UF-A7JC | 0.00054886 | 8.36E-05 |
| TCGA-QK-A6II | 0.0002073 | 4.00E-05 |
| TCGA-CN-A49B | 1.48E-05 | 2.90E-06 |
| TCGA-IQ-A61E | 5.26E-05 | 4.32E-06 |
| TCGA-CV-7101 | 0.00015909 | 3.99E-07 |
| TCGA-BA-A4IH | 5.61E-06 | 3.20E-07 |
| TCGA-H7-8502 | 0.00028805 | 2.28E-05 |
| TCGA-CV-7414 | 0.00010915 | 4.42E-06 |
| TCGA-HD-7229 | 0.00029407 | 2.57E-05 |
| TCGA-CV-6961 | 6.26E-05 | 5.03E-06 |
| TCGA-TN-A7HI | 3.79E-05 | 1.49E-05 |
| TCGA-CQ-5333 | 5.29E-05 | 5.65E-06 |
| TCGA-CN-4733 | 0.00011409 | 3.31E-05 |
| TCGA-MT-A67F | 9.80E-05 | 1.91E-07 |
| TCGA-CR-7385 | 0.00027548 | 2.55E-05 |
| TCGA-TN-A7HJ | 4.84E-05 | 1.45E-05 |
| TCGA-CN-4736 | 0.00011619 | 1.05E-05 |
| TCGA-CN-4730 | 4.72E-05 | 1.15E-05 |
| TCGA-CV-7433 | 0.00010967 | 6.60E-07 |
| TCGA-CN-4726 | 0.00018768 | 3.43E-05 |
| TCGA-CQ-5332 | 0.0001 | 6.49E-06 |
| TCGA-HD-8224 | 0.00014099 | 1.25E-05 |
| TCGA-BB-8601 | 5.31E-05 | 1.30E-05 |
| TCGA-CN-5355 | 0.00044785 | 9.72E-05 |
| TCGA-QK-A6IG | 6.78E-05 | 7.07E-06 |
| TCGA-D6-6826 | 4.32E-05 | 6.45E-06 |
| TCGA-CR-7404 | 0.00038613 | 6.13E-05 |
| TCGA-D6-A6EM | 0.00017833 | 3.87E-05 |
| TCGA-CQ-A4CE | 0.00029773 | 9.68E-05 |
| TCGA-CV-A460 | 7.39E-05 | 9.72E-08 |
| TCGA-CV-7104 | 0.00020668 | 2.52E-05 |
| TCGA-UF-A7JF | 8.81E-05 | 1.21E-05 |
| TCGA-HD-7753 | 0.00012325 | 3.02E-05 |
| TCGA-F7-A61S | 0.0001025 | 1.03E-05 |
| TCGA-CN-6017 | 3.93E-05 | 7.64E-06 |
| TCGA-CR-7369 | 0.00021316 | 1.67E-05 |
| TCGA-P3-A6T8 | 5.29E-05 | 1.61E-07 |
| TCGA-F7-A50J | 0.00024483 | 8.81E-06 |
| TCGA-CV-7100 | 6.12E-05 | 6.73E-06 |
| TCGA-TN-A7HL | 1.26E-05 | 1.34E-06 |
| TCGA-CV-7090 | 0.00011875 | 1.17E-05 |
| TCGA-BA-6871 | 4.92E-05 | 2.26E-06 |
| TCGA-CR-7371 | 5.24E-05 | 1.09E-07 |
| TCGA-CV-5432 | 0.00018775 | 1.35E-05 |
| TCGA-CN-5360 | 0.00053563 | 0.00010862 |
| TCGA-CQ-A4C6 | 9.75E-05 | 1.73E-05 |
| TCGA-MT-A7BN | 0.00016872 | 2.80E-05 |
| TCGA-CN-A641 | 0.00010584 | 7.77E-07 |
| TCGA-CQ-A4C9 | 0.00048963 | 0.000128812 |
| TCGA-CQ-A4CA | 8.96E-05 | 7.91E-06 |
| TCGA-IQ-A61O | 0.00129389 | 6.88E-05 |
| TCGA-CQ-A4CD | 3.33E-05 | 6.13E-06 |
| TCGA-CN-A499 | 6.97E-05 | 1.75E-05 |
| TCGA-CN-6013 | 0.00012548 | 3.49E-07 |
| TCGA-CN-5356 | 0.00015017 | 8.83E-06 |
| TCGA-CV-7242 | 5.80E-05 | 1.32E-05 |
| TCGA-CN-5369 | 0.00030696 | 6.28E-05 |
| TCGA-CN-6988 | 0.00010897 | 9.14E-06 |
| TCGA-D6-A6EN | 2.14E-05 | 4.29E-06 |
| TCGA-CQ-A4C7 | 6.17E-05 | 4.40E-07 |
| TCGA-CV-5435 | 9.76E-05 | 2.86E-06 |
| TCGA-CR-7370 | 7.07E-05 | 6.73E-06 |
| TCGA-CN-4722 | 0.00062616 | 3.22E-05 |
| TCGA-CN-6023 | 6.36E-05 | 5.03E-08 |
| TCGA-CQ-A4CI | 7.25E-05 | 8.52E-06 |
| TCGA-CV-7438 | 0.0001131 | 2.09E-05 |
| TCGA-BB-4224 | 6.81E-05 | 5.02E-06 |
| TCGA-CN-6997 | 1.81E-05 | 2.95E-07 |
| TCGA-CV-6937 | 0.00011667 | 7.06E-06 |
| TCGA-CQ-5326 | 0.00028176 | 2.94E-05 |
| TCGA-BA-A6DG | 5.70E-05 | 1.61E-05 |
| TCGA-UF-A7JT | 4.87E-05 | 4.06E-06 |
| TCGA-CN-A6V6 | 4.63E-05 | 5.89E-08 |
| TCGA-CR-6473 | 6.28E-05 | 6.71E-06 |
| TCGA-BA-7269 | 4.62E-05 | 1.56E-07 |
| TCGA-CV-7177 | 3.01E-05 | 8.68E-06 |
| TCGA-BB-A6UO | 0.00014287 | 1.06E-05 |
| TCGA-CV-A468 | 0.00033468 | 8.50E-05 |
| TCGA-CQ-5324 | 8.42E-05 | 8.02E-06 |
| TCGA-CQ-A4CH | 0.00012467 | 1.02E-05 |
| TCGA-CN-6020 | 0.00011419 | 1.78E-05 |
| TCGA-CN-A63V | 0.00013519 | 1.44E-05 |
| TCGA-CV-7415 | 4.55E-05 | 4.07E-07 |
| TCGA-CV-6003 | 0.00015752 | 2.55E-05 |
| TCGA-CN-6016 | 0.00045674 | 1.91E-05 |
| TCGA-CV-5966 | 3.35E-05 | 4.37E-06 |
| TCGA-BA-4076 | 0.00011718 | 1.25E-05 |
| TCGA-CN-4735 | 3.37E-05 | 8.22E-08 |
| TCGA-CV-6955 | 0.00030966 | 7.97E-05 |
| TCGA-CR-6491 | 0.00091828 | 8.58E-05 |
| TCGA-BA-5558 | 0.0002006 | 3.66E-06 |
| TCGA-CN-4728 | 2.49E-05 | 2.58E-06 |
| TCGA-BB-A5HZ | 0.00010428 | 7.63E-06 |
| TCGA-UP-A6WW | 4.10E-05 | 1.55E-07 |
| TCGA-CV-7407 | 0.00013204 | 5.19E-06 |
| TCGA-CV-6936 | 0.00015228 | 9.70E-06 |
| TCGA-CV-7416 | 6.50E-05 | 2.07E-05 |
| TCGA-CN-5361 | 6.57E-05 | 2.13E-05 |
| TCGA-CN-A49A | 8.97E-05 | 8.02E-06 |
| TCGA-CN-6998 | 0.00048638 | 1.50E-05 |
| TCGA-MZ-A6I9 | 4.78E-05 | 5.77E-06 |
| TCGA-F7-A50I | 0.00014569 | 8.07E-07 |
| TCGA-T2-A6WX | 2.96E-05 | 2.47E-06 |
| TCGA-CQ-6229 | 0.00020237 | 1.43E-05 |
| TCGA-CN-A63U | 1.90E-05 | 2.07E-06 |
| TCGA-CV-6940 | 3.97E-05 | 9.84E-06 |
| TCGA-H7-8501 | 3.70E-05 | 1.32E-06 |
| TCGA-CV-7255 | 6.36E-05 | 2.35E-05 |
| TCGA-CR-5243 | 3.20E-05 | 8.05E-08 |
| TCGA-P3-A6T5 | 0.00019399 | 1.58E-05 |
| TCGA-CV-6952 | 0.00026609 | 5.75E-05 |
| TCGA-D6-A6ES | 0.00057709 | 7.24E-05 |
| TCGA-UF-A7J9 | 0.00013131 | 1.25E-06 |
| TCGA-CQ-6225 | 6.28E-05 | 4.94E-06 |
| TCGA-MT-A51X | 9.27E-05 | 1.57E-06 |
| TCGA-BA-5152 | 3.49E-05 | 1.47E-06 |
| TCGA-CQ-7065 | 0.00011495 | 3.18E-05 |
| TCGA-CN-4741 | 0.00019533 | 1.69E-05 |
| TCGA-CR-7380 | 3.02E-05 | 1.52E-07 |
| TCGA-F7-A61W | 2.70E-05 | 3.90E-06 |
| TCGA-CV-5439 | 3.63E-05 | 3.95E-06 |
| TCGA-HD-A4C1 | 0.00011161 | 2.39E-05 |
| TCGA-D6-6517 | 0.0002516 | 1.56E-05 |
| TCGA-CQ-6227 | 7.17E-05 | 4.49E-06 |
| TCGA-CV-7089 | 0.00018968 | 3.17E-05 |
| TCGA-QK-AA3K | 6.76E-05 | 3.26E-06 |
| TCGA-CN-5367 | 0.00011892 | 2.50E-05 |
| TCGA-CN-5365 | 0.00020514 | 5.47E-05 |
| TCGA-CV-7095 | 0.00026223 | 3.09E-05 |
| TCGA-QK-A8ZA | 8.93E-05 | 2.90E-07 |
| TCGA-CV-A6JE | 6.73E-05 | 8.66E-06 |
| TCGA-CQ-7063 | 6.66E-05 | 3.64E-07 |
| TCGA-BB-4227 | 8.06E-05 | 2.73E-06 |
| TCGA-CV-6433 | 2.29E-05 | 8.88E-08 |
| TCGA-CN-5373 | 0.00054849 | 6.33E-05 |
| TCGA-CV-7568 | 7.23E-05 | 9.68E-06 |
| TCGA-CN-6989 | 5.06E-05 | 6.86E-07 |
| TCGA-CV-7424 | 0.00056701 | 0.000211966 |
| TCGA-BA-4078 | 8.79E-05 | 3.33E-07 |
| TCGA-CN-4739 | 8.80E-05 | 4.22E-06 |
| TCGA-CV-5430 | 6.16E-05 | 2.07E-07 |
| TCGA-P3-A6SX | 0.00013175 | 6.09E-06 |
| TCGA-CV-7252 | 9.52E-05 | 7.02E-06 |
| TCGA-D6-A6EQ | 0.00035157 | 2.90E-05 |
| TCGA-CN-6995 | 6.59E-05 | 9.46E-06 |
| TCGA-KU-A6H7 | 3.54E-05 | 6.57E-06 |
| TCGA-D6-A6EO | 0.00010875 | 5.73E-06 |
| TCGA-KU-A6H8 | 3.21E-05 | 2.08E-07 |
| TCGA-CN-6010 | 2.55E-05 | 5.23E-07 |
| TCGA-BA-A8YP | 3.73E-05 | 6.82E-07 |
| TCGA-D6-A4ZB | 9.23E-05 | 6.30E-06 |
| TCGA-CV-7422 | 4.78E-05 | 2.16E-06 |
| TCGA-CX-A4AQ | 1.75E-05 | 1.30E-07 |
| TCGA-CQ-5330 | 0.00010087 | 1.55E-05 |
| TCGA-P3-A6T3 | 0.00018945 | 2.84E-05 |
| TCGA-BB-A5HY | 4.98E-05 | 2.35E-06 |
| TCGA-CV-A45R | 6.38E-05 | 1.70E-05 |
| TCGA-P3-A6T4 | 0.00018041 | 8.65E-06 |
| TCGA-MT-A51W | 0.00015512 | 3.73E-05 |
| TCGA-CQ-7068 | 4.07E-05 | 6.34E-06 |
| TCGA-4P-AA8J | 1.50E-05 | 9.80E-07 |
| TCGA-CV-A45P | 0.00015166 | 8.50E-06 |
| TCGA-CV-7429 | 2.34E-05 | 5.25E-08 |
| TCGA-HD-A633 | 9.77E-05 | 1.23E-05 |
| TCGA-DQ-5631 | 0.00017768 | 9.94E-06 |
| TCGA-CV-5436 | 0.00015035 | 1.90E-05 |
| TCGA-DQ-5630 | 5.79E-05 | 1.84E-06 |
| TCGA-CR-6482 | 2.30E-05 | 1.15E-06 |
| TCGA-P3-A6T6 | 4.88E-05 | 3.29E-06 |
| TCGA-DQ-7592 | 0.00023104 | 1.33E-06 |
| TCGA-BA-A4II | 6.51E-05 | 1.23E-05 |
| TCGA-HL-7533 | 2.90E-05 | 1.43E-06 |
| TCGA-D6-6516 | 0.00061024 | 9.59E-05 |
| TCGA-CV-A45V | 4.76E-05 | 5.72E-06 |
| TCGA-H7-7774 | 0.00033238 | 4.98E-05 |
| TCGA-CQ-A4CG | 7.51E-05 | 1.19E-05 |
| TCGA-CV-5970 | 0.00016138 | 8.08E-07 |
| TCGA-BB-4217 | 1.72E-05 | 2.17E-06 |
| TCGA-CR-5248 | 0.00011699 | 3.31E-05 |
| TCGA-CR-7394 | 0.00107355 | 0.000424617 |
| TCGA-CV-7421 | 4.86E-05 | 1.04E-05 |
| TCGA-CN-4729 | 0.00026768 | 3.30E-05 |
| TCGA-T2-A6WZ | 0.00063733 | 0.000315463 |
| TCGA-CV-6436 | 0.0002274 | 8.46E-07 |
| TCGA-UF-A7JA | 0.00011117 | 1.18E-07 |
| TCGA-CQ-A4CB | 8.46E-05 | 1.19E-06 |
| TCGA-CX-7219 | 4.27E-05 | 9.04E-06 |
| TCGA-IQ-A61G | 0.00015942 | 9.20E-06 |
| TCGA-CV-5434 | 1.99E-05 | 3.42E-06 |
| TCGA-RS-A6TP | 7.05E-05 | 1.64E-06 |
| TCGA-CV-5978 | 4.11E-05 | 1.47E-07 |
| TCGA-CV-5977 | 0.00026212 | 1.71E-05 |
| TCGA-CV-A6JU | 1.45E-05 | 9.70E-07 |
| TCGA-CV-7102 | 0.00011617 | 2.35E-05 |
| TCGA-CR-7368 | 0.00012929 | 1.57E-07 |
| TCGA-D6-A74Q | 0.0004642 | 0.000143187 |
| TCGA-D6-6824 | 1.68E-05 | 4.60E-08 |
| TCGA-CV-5444 | 9.63E-05 | 3.05E-06 |
| TCGA-CN-A6V7 | 4.89E-05 | 6.89E-06 |
| TCGA-CV-A45Z | 4.88E-05 | 7.13E-06 |
| TCGA-BA-A6DE | 0.00014406 | 3.63E-06 |
| TCGA-D6-8569 | 0.00017911 | 9.82E-06 |
| TCGA-CR-7401 | 0.00016515 | 5.07E-06 |
| TCGA-CV-7247 | 7.37E-05 | 1.00E-05 |
| TCGA-CQ-7072 | 4.42E-05 | 9.22E-06 |
| TCGA-CV-A45T | 2.81E-05 | 3.49E-07 |
| TCGA-BA-4075 | 1.99E-05 | 3.31E-06 |
| TCGA-DQ-5624 | 0.0001869 | 1.58E-05 |
| TCGA-BA-A6DD | 4.37E-05 | 4.97E-06 |
| TCGA-KU-A66S | 1.51E-05 | 2.07E-06 |
| TCGA-CV-7254 | 0.00052274 | 0.000195934 |
| TCGA-CV-5976 | 6.62E-05 | 1.78E-05 |
| TCGA-CR-7374 | 5.64E-05 | 1.13E-05 |
| TCGA-CR-5250 | 5.12E-05 | 8.38E-06 |
| TCGA-CV-A6JZ | 6.88E-05 | 9.42E-06 |
| TCGA-CV-6954 | 7.41E-05 | 2.58E-06 |
| TCGA-P3-A5QE | 3.80E-05 | 5.98E-06 |
| TCGA-QK-A8Z8 | 6.87E-05 | 1.68E-06 |
| TCGA-CV-A465 | 0.00052526 | 5.52E-05 |
| TCGA-DQ-5629 | 0.0001368 | 1.43E-05 |
| TCGA-BA-A6DJ | 5.47E-05 | 9.29E-06 |
| TCGA-CR-7395 | 0.00012967 | 6.75E-07 |
| TCGA-CQ-6223 | 0.00023167 | 4.69E-05 |
| TCGA-F7-7848 | 3.17E-05 | 1.22E-07 |
| TCGA-CV-A6JM | 0.00033537 | 4.03E-05 |
| TCGA-CN-5359 | 7.52E-05 | 2.31E-06 |
| TCGA-P3-A5QA | 0.00011838 | 1.28E-05 |
| TCGA-CV-6948 | 4.55E-05 | 1.37E-06 |
| TCGA-QK-A64Z | 0.00010467 | 1.14E-05 |
| TCGA-CQ-6228 | 7.28E-05 | 1.19E-05 |
| TCGA-CN-6996 | 0.00036118 | 4.67E-05 |
| TCGA-CV-5431 | 9.74E-05 | 3.89E-05 |
| TCGA-CV-A45O | 0.0005715 | 0.000107012 |
| TCGA-T3-A92N | 6.82E-05 | 6.55E-06 |
| TCGA-CR-7399 | 1.43E-05 | 6.73E-07 |
| TCGA-CQ-5334 | 0.0001086 | 3.15E-07 |
| TCGA-CR-7402 | 5.62E-05 | 3.51E-07 |
| TCGA-CR-7389 | 6.40E-05 | 9.79E-06 |
| TCGA-BA-6868 | 0.00011137 | 1.79E-05 |
| TCGA-CR-6488 | 0.00017422 | 1.94E-05 |
