# Supplementary figures and images for: A novel immune signature predicts immunotherapy responsiveness and reveals the landscape of the tumor immune microenvironment in head and neck squamous cell carcinoma
Source: Front Genet. 2022 Nov 11;13:1051051. doi: 10.3389/fgene.2022.1051051 (PMC9691887; doi:10.3389/fgene.2022.1051051)

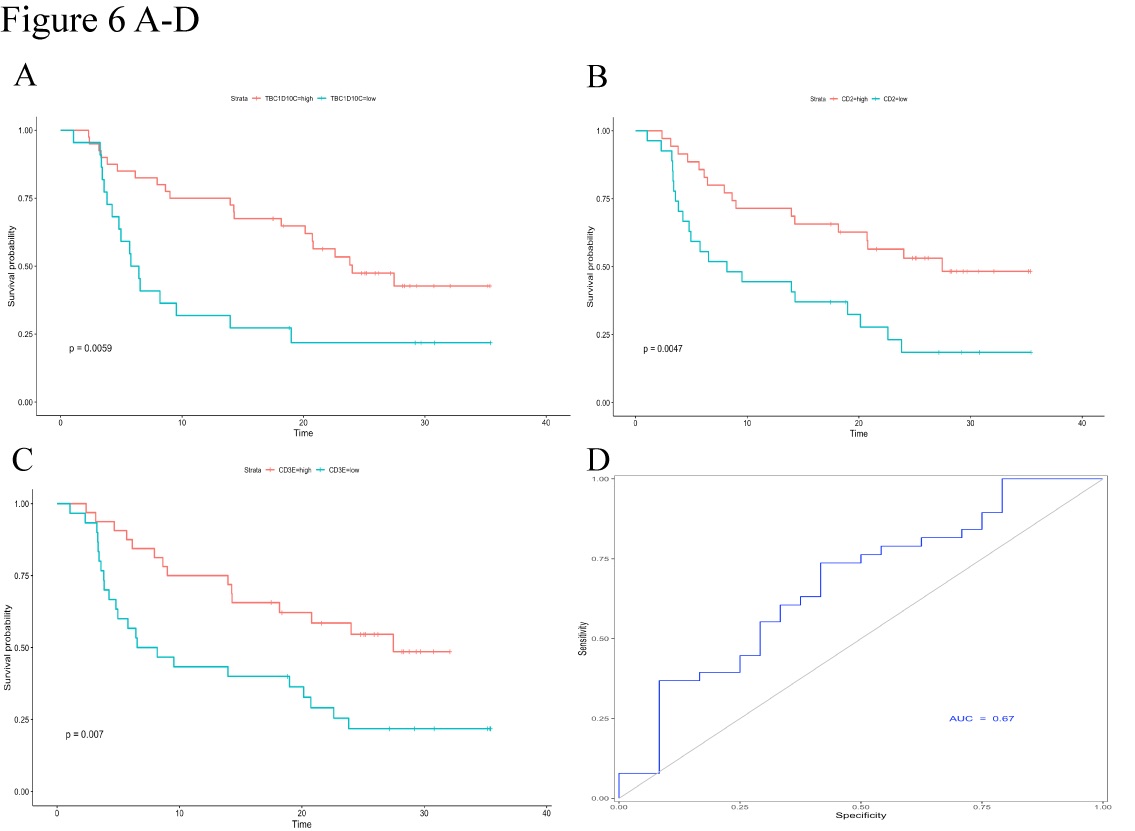

Supplement: Supplementary file 4 [file Image1.JPEG]
